# Impact of diagnostic accuracy on the estimation of excess mortality from incidence and prevalence: simulation study and application to diabetes in German men

**DOI:** 10.12688/f1000research.28023.1

**Published:** 2021-01-27

**Authors:** Ralph Brinks, Thaddäus Tönnies, Annika Hoyer

**Affiliations:** 1 Institute for Biometry and Epidemiology, German Diabetes Center, Duesseldorf, 40225, Germany; 2 Chair for Medical Biometry and Epidemiology, Faculty of Health/School of Medicine, Witten/Herdecke University, Witten, 58448, Germany; 3 Department of Statistics, Ludwig Maximilian University of Munich, Munich, 80539, Germany

**Keywords:** Illness-death model, chronic conditions, diabetes, lupus, partial differential equations, epidemiology

## Abstract

Aggregated data about the prevalence and incidence of chronic conditions is becoming more and more available. We recently proposed a method to estimate the age-specific excess mortality in chronic conditions from aggregated age-specific prevalence and incidence data. Previous works showed that in age groups below 50 years, estimates from this method were unstable or implausible. In this article, we examine how limited diagnostic accuracy in terms of sensitivity and specificity affects the estimates. We use a simulation study with two settings, a low and a high prevalence setting, and assess the relative importance of sensitivity and specificity. It turns out that in both settings, specificity, especially in the younger age groups, dominates the quality of the estimated excess mortality. The findings are applied to aggregated claims data comprising the diagnoses of diabetes from about 35 million men in the German Statutory Health Insurance. Key finding is that specificity in the lower age groups (<50 years) can be derived without knowing the sensitivity. The false-positive ratio in the claims data increases linearly from 0.5 per mil at age 25 to 2 per mil at age 50.

As a conclusion, our findings stress the importance of considering diagnostic accuracy when estimating excess mortality from aggregated data using the method to estimate excess mortality. Especially the specificity in the younger age-groups should be carefully taken into account.

## Introduction

For research purposes, aggregated data about the prevalence and incidence of chronic conditions become more and more available. Examples range from data of huge public health surveys, such as the National Health Interview Study (NHIS) in the US [ CDC 2020] or the Global Health Data Exchange (GHDx) catalog [ GHD 2020], which covers up to three decades of international health data, to claims data from health service providers [ CMS 2020].

Recently, we proposed a new method to estimate the age-specific excess mortality in chronic conditions from aggregated age-specific prevalence and incidence data based on a differential equation [ Tönnies *et al.*, 2018; Brinks *et al.*, 2019]. The idea, in brief, is to relate the temporal change of the prevalence with the incidence and the excess mortality. If the incidence and prevalence are given, the excess mortality can be estimated. In age groups below 50 years of age, estimates from this method have been proven to be unstable or implausible [ Brinks *et al.*, 2020]. For example, we obtained estimates of the mortality rate ratio in type 2 diabetes with values greater than 100 in ages below 40 years [ Brinks *et al.*, 2020]. The typical range for type 2 diabetes in this age group is between 3 and 10 [ Carstensen *et al.*, 2020]. In [ Brinks *et al.*, 2020] it was hypothesized “that the diagnostic accuracy of the claims data plays a crucial role for the proposed methods of estimating excess mortality.”

Similar to diagnostic accuracy studies, we are interested in the sensitivity and specificity of the available diagnoses in the claims data. As “gold standard” we consider the presence or absence of the chronic condition in real life (as judged by an expert from the associated medical domain). Within the claims data, two types of error may occur: People with the condition in real life might not have the diagnosis coded in the claims data (false negative) or vice versa, people without the condition in real life might have a corresponding diagnosis (false positive). Finally, this leads to the concept of sensitivities and specificities of the aggregated prevalence and incidence data.

The aim of this article is twofold: First, we want to examine and quantify the impact of diagnostic accuracy on the estimates of excess mortality. For this, we use a simulation study comprising two settings, a low and high prevalence setting. Second, as a real-world application of the findings in the first part, we estimate the age-specific diagnostic accuracy of claims data about diabetes from about 35 million German men in the Statutory Health Insurance [ Goffrier *et al.*, 2017].

## Methods

Before we start with the simulation and the real-world application, we briefly sketch the theoretical background. Detailed derivations are given in *Extended Data* [ Brinks *et al.*, 2021].

**Figure 1.**
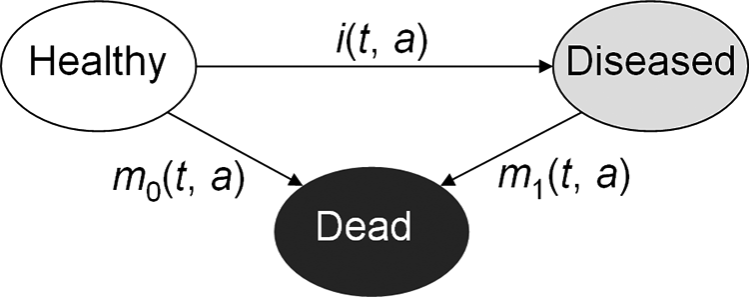
Illness-death model. People aged *a* at time *t* in the population are in one of the three states: *Healthy*, *Diseased*, or *Dead.* Transitions between these states are described by the rates *i*, *m*_0_, and *m*_1_, which in general depend on *t* and *a.*

Based on the illness-death model for chronic diseases ( Figure 1), it can be shown that the temporal change, $\partial p=\left(\partial_{t}+\partial_{a}\right)\;p$ , of the age-specific prevalence *p* is related to the incidence rate *i*, and the mortality rates *m*_0_ and *m*_1_ of the people with and without the chronic condition (disease), respectively. Instead of the rates *m*_0_ and *m*_1_, the general mortality *m* = *pm*_1_ + (1 − *p*) *m*_0_ and the mortality rate ratio *R* = *m*_1_/ *m*_0_ can be used according to the following equations [ Brinks *et al.*, 2014; Brinks *et al.*, 2016]:

(1) $$\begin{array}{ll} \partial p & =(1-p)\{i-p\times (m_{1}-m_{0})\}\\ & =(1-p)\{i-m\times p(R-1)/[1+p(R-1)]\}. \end{array}$$

Given the age-specific prevalence *p*, the age-specific incidence rate *i* and the general mortality rate *m*, Equation (1) provides an estimator for the mortality rate ratio *R*:

(2) R=1+1/p×{i−∂p/(1−p)}/{m−i+∂p/(1−p)}.

Assuming that the sensitivity ( *se*) and specificity ( *sp*) in the age-specific prevalence and incidence are known, the prevalence *p* and incidence *i* in Equations (1) and (2) can be obtained from the observed (and possibly imperfect) prevalence *p* ^(obs)^ and incidence *i* ^(obs)^ by

(3a) $$p=\left(p^{\left(\mathrm{obs}\right)}-1+sp_{p}\right)/\left(se_{p}+sp_{p}-1\right)$$

and

(3b) $$i=\left(i^{\left(\mathrm{obs}\right)}-1+sp_{i}\right)/\left(se_{i}+sp_{i}-1\right).$$

The derivations of these equations are shown in Extended Data Appendix 2 [ Brinks *et al.*, 2021]. The observed values *p* ^(obs)^ and *i* ^(obs)^ may have been prone to error by incomplete case-detection (i.e., *se* < 1) and/or false positive findings ( *sp* < 1). If all sensitivities and specificities equal 1, we find *p* = *p* ^(obs)^ and *i* = *i* ^(obs)^. Note that in Equations (3a) and (3b) we distinguish between sensitivities and specificities in prevalence and incidence (indicated by the sub-indices *p* and *i*, respectively). To examine potential age effects, *se* and *sp* may depend on age *a.* Age dependency is taken into account, because diagnostic accuracy in many diseases is known to depend on age. For example, sensitivity of diagnosing type 2 diabetes in 80 years old people is higher than in 40 year old people, which is, for instance, reflected by the higher percentage of undiagnosed diabetes in younger age groups [ Gregg *et al.*, 2004].

### Simulation studies

The steps for running the simulation studies in the low and high prevalence setting are as follows: We first solve Equation (1) with known *i*, *m* and *R* to obtain prevalence data *p.* Second, imperfect diagnostic accuracy is mimicked by using Equations (3a) and (3b) such that the quantities *p* ^(obs)^ and *i* ^(obs)^ are observed instead of the (true) quantities *p* and *i.* In the third step, Equation (2) is applied to *p* ^(obs)^ and *i* ^(obs)^ in order to obtain an estimate for the mortality rate ratio ( *R* ^(obs)^). Finally, *R* ^(obs)^ is compared to the true *R* underlying the simulation. This is done for a wide range of age-groups ( Table 1).

**Table 1.** Description of the parameter settings in the simulations.

	Setting	
	Low prevalence	High prevalence
Incidence *i*	Lupus in women [ Brinks *et al.*, 2016]	Type 2 diabetes in men [ Tamayo *et al.*, 2016]
Mortality rate ratio *R*	Lupus [ Bernatsky *et al.*, 2006]	Type 2 diabetes [ Carstensen *et al.*, 2020]
General mortality *m*	Federal Statistical Office of Germany [ FSG 2020]	Federal Statistical Office of Germany [ FSG 2020]
Considered age range	20-70 years	40-80 years
Sensitivity (base-case) younger age older age	99.5% at 20 years of age 99.5% at 70 years of age	95% at 40 years of age 95% at 80 years of age
Specificity (base-case) younger age older age	99.999% at 20 years of age 99.999% at 70 years of age	99.95% at 40 years of age 99.95% at 80 years of age

We use two figures for the comparisons: 1) The age-specific difference between *R* and *R* ^(obs)^ and 2) the summed absolute relative errors (where the sum is taken over the whole considered age range). The later figure is used to assess the relative importance of the sensitivities and specificities in the form of a tornado plot. A tornado plot displays the change of the considered outcome compared to a base-case scenario, if exactly one input variable, say the sensitivity of the incidence in an age group, is changed while all the other input values (i.e., the remaining sensitivities and specificities) are kept fixed. This is done for all input variables. The changes in the output are presented as vertical bars, which are then ordered descendingly to indicate the importance of the associated input variables on the output. The descending order leads to the largest bar being presented on top and the smallest bar at the bottom, which visually appears as a half of a tornado (see Figure 3).

Table 1 shows the parameters for the two simulation settings in the low and the high prevalence scenarios. The low and the high prevalence scenarios are motivated by systemic lupus erythematosus (SLE) in women and type 2 diabetes in men, respectively. As SLE is more relevant in younger ages, we consider the age range from 20 to 70 years in this setting. Type 2 diabetes is especially important for ages greater then 40, which lead us to the choice of considering the range 40 to 80 years of age. Although the values for the sensitivity and specificity in Table 1 are the same in the younger and older ages, they are treated independently to allow exploration of the relative importance in the tornado plots. In any case, sensitivities and specificities are interpolated affine-linearly between the younger and the older age.

The source code for use with the free, open-source statistical software R (The R Foundation For Statistical Computing) can be found in [ Brinks *et al.*, 2020].

### Real world data

Based on claims data of German men in the Statutory Health Insurance (SHI), Goffrier and colleagues report the age-specific prevalence *p* ^(obs)^ of type 2 diabetes in the years 2009 and 2015 [ Goffrier *et al.*, 2017]. Furthermore, the age- and sex-specific incidence rate *i* ^(obs)^ in middle of the period, i.e., in the year 2012, is given in the same report. In addition to the prevalence and incidence, the mortality rate ratios *R* of men with and without diabetes in the German SHI in the year 2014 have been reported in [ Scheidt-Nave 2019]. Strictly speaking, the estimates of *R* from [ Scheidt-Nave 2019] might have undergone diagnostic inaccuracies as well. However, the estimates are based on individual data (ID) and potential biases in ID analyses (e.g., by missing disease status at death [ Binder *et al.*, 2017]), are beyond the scope of this article. Thus, for simplicity we assume *R* = *R* ^(obs)^.

We use these data about *p* ^(obs)^, *i* ^(obs)^ and *R* to obtain estimates about the age-specific sensitivity and specificity of the prevalence and incidence via Equations (3a) and (3b). For this, we make the following approach: for each age group (denoted *a*_k_, *k* = 1, …, *K*) we assume that the sensitivity and specificity of prevalence and incidence are the same, i.e., *se*_p_( *a*_k_) *= se*_i_( *a*_k_) and *sp*_p_( *a*_k_) = *sp*_i_( *a*_k_), for all *k* = 1, …, *K.* The assumption of same sensitivity and specificity with respect to prevalence and incidence is justified because prevalent and incident cases are derived from reported diagnoses of all physicians treating the men in the SHI. If prevalence data suffer from incomplete case-detection or false positive findings, incidence data will suffer in the same way.

If we assume for the moment that the sensitivity *se* = *se*_p_ *= se*_i_ is known, we can combine Equations (3a) and (3b) with Equation (1) to estimate the specificity *sp* = *sp*_p_ *= sp*_i_. This is possible, because with given general mortality *m* from the Federal Statistical Office of Germany [ FSG 2020], all measures *p* ^(obs)^, *i* ^(obs)^, and *R* in Equation (1) are known from [ Goffrier *et al.*, 2017] and [ Scheidt-Nave 2019] after applying the corrections in Equations (3a) and (3b). Hence for known sensitivity *se*, we can calculate *sp* from these data and the analytical findings in the previous section by a functional relation Φ

(4) $$sp=\Phi \left(se,p^{\left(\mathrm{obs}\right)},i^{\left(\mathrm{obs}\right)},m,R\right)$$

The exact formula for the functional relation Φ between *sp* on the left hand side and *se*, *p* ^(obs)^, *i* ^(obs)^, *m*, and *R* on the right hand side of Equation (4), is lengthy and presented together with its derivation and an algorithm in Extended Data Appendix 3 [ Brinks *et al.*, 2021]. An implementation of the algorithm in the statistical software R can be found in [ Brinks *et al.*, 2020]. For now, it is sufficient to notice that the relation in Equation (4) follows from Equations (1), (3a) and (3b).

Unfortunately, we do not know the sensitivity of the diagnoses in the claims data. To overcome this problem, we use a probabilistic approach and randomly sample *se* from epidemiologically reasonable ranges between 70% and 99%. Then, we examine how the estimated specificity *sp* changes. For easier interpretation, we present the false positive ratio (FPR), FPR = 1 − *sp.*

The data and the source code for use with the free statistical software R (The R Foundation For Statistical Computing) can be found in [ Brinks *et al.*, 2020] (DOI: 10.5281/zenodo.4300684).

## Results

### Simulation studies

**Figure 2.**
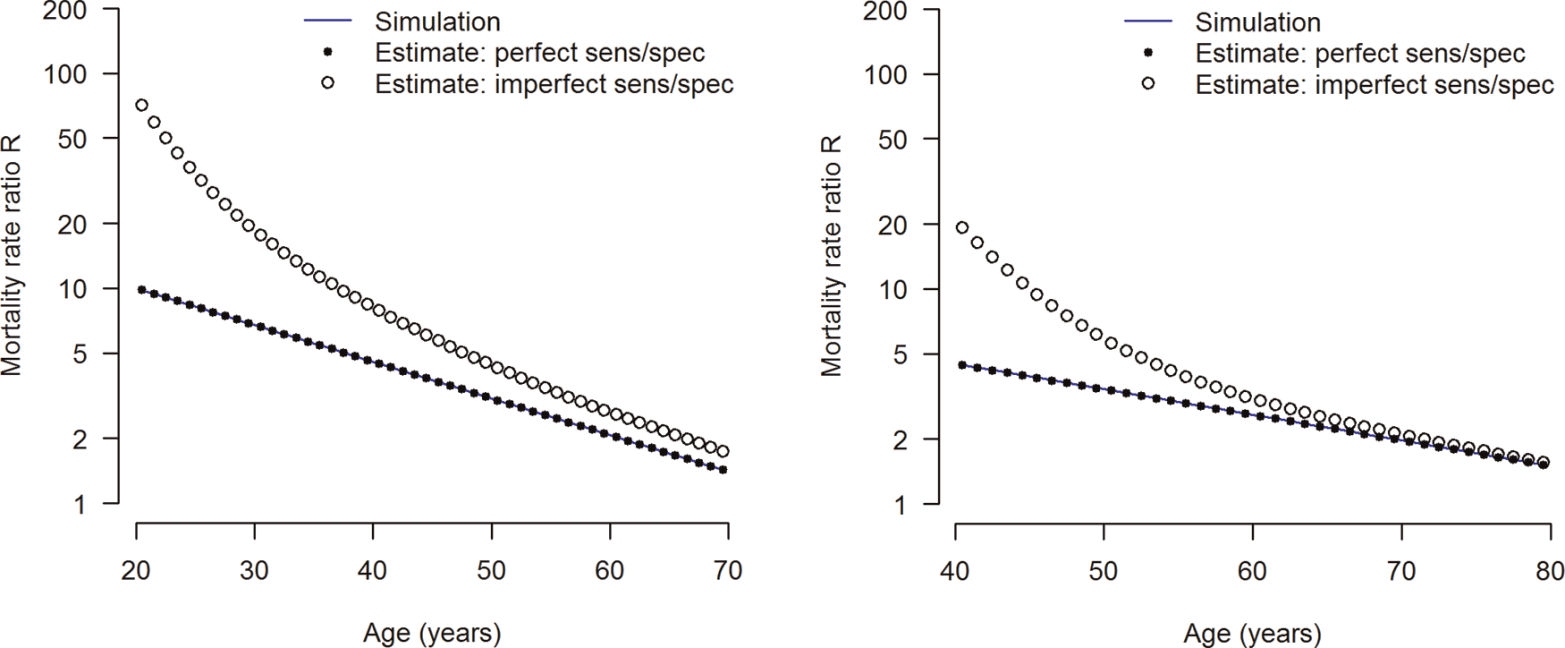
Age-specific mortality rate ratios ( *R*) in the simulations. The low prevalence and high prevalence setting are shown in the left and right panels, respectively. The input values are shown as blue lines. Mortality rate ratios *R* are estimated without any (visual) difference in case of perfect sensitivity *se* = 100% and perfect specificity *sp* = 100% (solid dots). In case of imperfect sensitivity and specificity, the estimates of *R* are biased upward (open circles).

Figure 2 shows the estimated age-specific mortality rate ratios *R* in the simulation studies. The left and right panel in Figure 2 refers to the low and high prevalence settings, respectively. While in case of perfect diagnostic accuracy, i.e. *sp* = *se* = 100%, the input values of the simulation (blue lines) and the estimates by Equation (2) (solid black dots) do not (visually) differ. Imperfect sensitivity and specificity lead to estimates biased upwards (open circles). It becomes visible that with increasing age the difference between the true and estimated values decreases.

**Figure 3.**
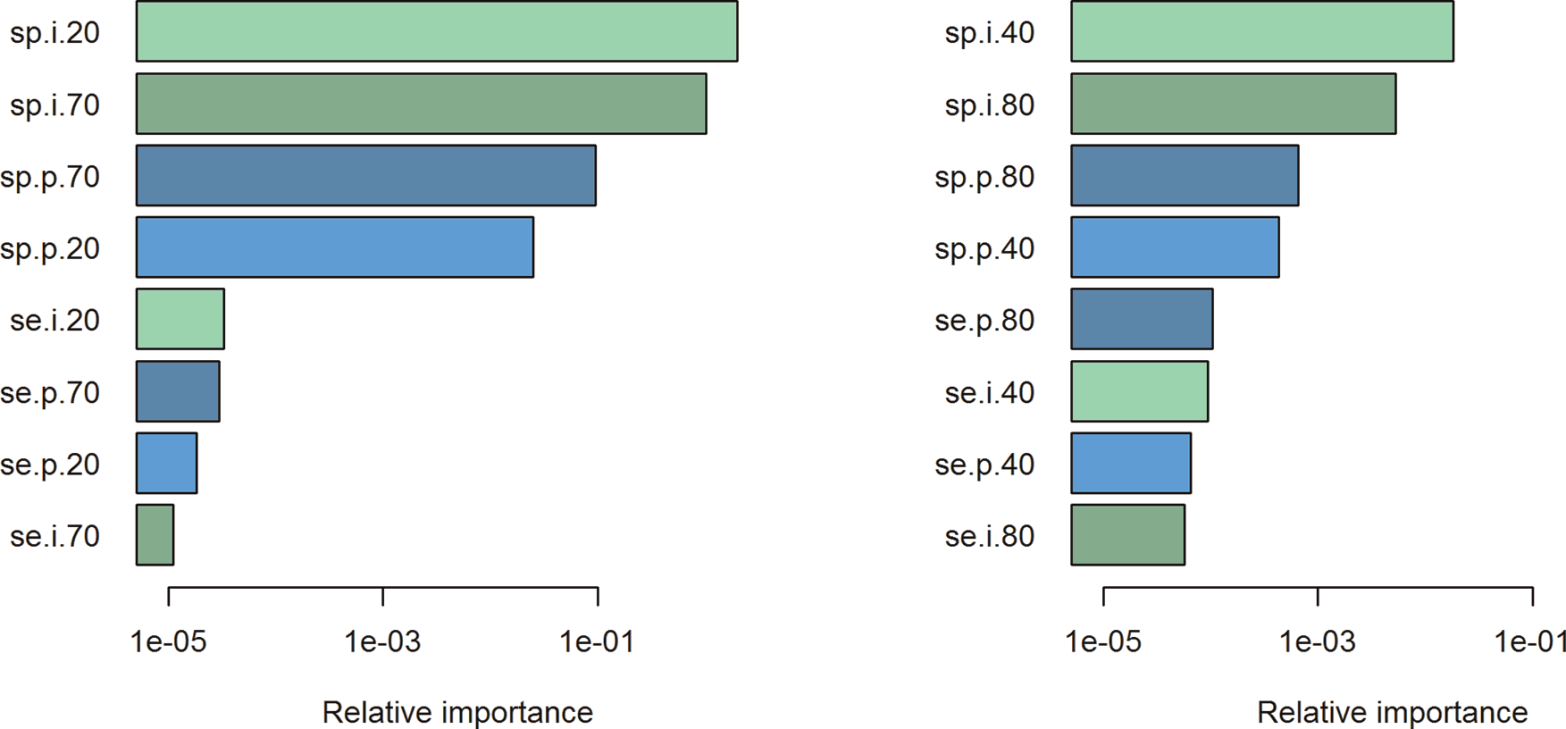
Tornado plots for relative importance of the sensitivity and specificity. In both settings, low (left panel) and high prevalence (right), the specificities (prefix sp) are the four dominant error factors in estimating the mortality rate ratio *R.* Compared to specificities, sensitivities (prefix se) have a low impact on the error in *R.*

In the assessment of the relative importance of the sensitivity and specificity in prevalence and incidence, we obtain the tornado plots as shown in Figure 3. Irrespective of the low (left panel in Figure 3) and high (right panel) prevalence setting, the specificity of the incidence ( *sp*_i_) in the lower age group has the greatest impact on the estimated mortality rate ratios. Specificity *sp*_i_ in the higher age group has the second strongest effect, followed by the specificities in prevalence ( *sp*_p_). The impact of the sensitivities is far weaker compared to the specificities. Note that the relative importance (abscissa) is given on the log scale.

By comparing the horizontal bars in the low and high prevalence settings, we see that the four specificities in the low prevalence settings have a greater effect than those in the high prevalence setting. The opposite is true in the sensitivities: in the high prevalence setting sensitivities have a larger impact than in the low prevalence setting.

### Real world data

**Figure 4.**
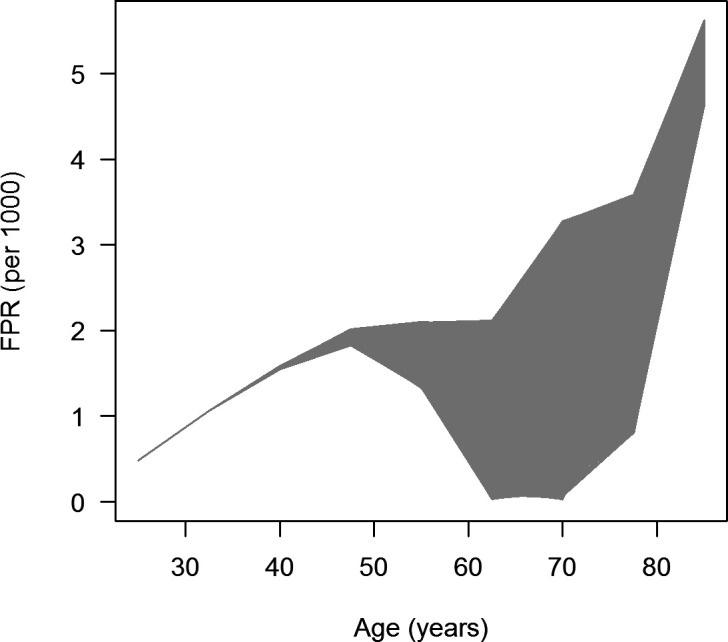
Age-specific false-positive ratios (FPR) in the simulated sensitivity scenarios. Each dot in the grey area represents the FPR generated by one of the scenarios about the age-specific sensitivities.

From Equation (4) we infer FPR = 1 - Φ( *se*, *p* ^(obs)^, *i* ^(obs)^, *m*, *R*). After uniformly sampling *se*( *a*_k_), where *a*_k_ = 25, 32.5, 40, …, 85, represents the *K* = 9 age groups [ *a*_k_ - 7.5/2, *a*_k_ + 7.5/2) of width 7.5 years, *k* = 1, …, 9, from the range 0.7 to 0.99 with *N* = 10000 samples, and calculating the associated FPR, we obtain the graph presented in Figure 4. Each dot in the grey area represents an FPR _n_( *a*_k_) based on a random *se*_n_( *a*_k_), *n* = 1, …, *N.* We see that irrespective of the randomly sampled values *se*_n_( *a*_k_) for *a*_k_ < 50, the FPR increases from 0.5 to 2 per mil. For example, at age 40 the FPR is about 1.5 per mil, which means that roughly 3 in 2000 diagnoses of type 2 diabetes at that age are false positive findings. For age groups > 50, we can see an upper bound for the FPR that continues linearly, while the lower bound can reach 0 at ages between 60 and 70 years. For higher ages, the lower bound of the FPR increases again.

## Discussion

In this work we have described the impact of diagnostic accuracy on the estimates of the excess mortality of a chronic condition from aggregated age-specific prevalence and incidence data. It turned out in simulation studies that the specificity in lower age groups had the greatest impact on the estimated mortality rate ratio. Compared to sensitivity, specificity has a greater impact across all age groups. The reason may be seen in the fact that the specificity has a direct additive effect on the true prevalence and incidence, while the sensitivity has an multiplicative impact only, cf. Equations (3a) and (3b).

In the simulation studies it turned out that estimation of the mortality rate ratio is accurately possible if the underlying sensitivity and specificities are known. In principle, these quantities are estimable in surveys. For example, in the claims data a cross-sectional comparison of the diagnoses with the gold standard (expert examination) could be conducted. These findings could be used to apply the corrections as in Equations (3a) and (3b) before using Equation (1) to estimate the mortality rate ratio.

By application of the theory to the claims data from 35 million German men, we were able to estimate the false positive ratio (FPR) in diabetes diagnoses. The most striking conclusion is the linearly increasing FPR in age groups between 20 and 50 years. In age groups older than 50 years of age, we could estimate upper and lower bounds for the FPR, which allows an assessment of diagnostic quality in the claims data.

Although most of our findings can be seen in the general theory of using the method of estimating excess mortality described in [ Tönnies *et al.*, 2018] and [ Brinks *et al.*, 2019], the application to real world data has two limitations that are important to mention. First, we assumed that the age-specific sensitivity and specificity are the same in both years 2009 and 2015. This might be an oversimplification, because it could, at least in principle, be that the diagnostic accuracy during this period of six years changed, for example, by implementation of screening programs, change of diagnostic criteria or by changes of reimbursement policies for diagnosing diabetes. However, we are not aware of such changes and refer studies about temporal changes in diagnostic accuracy to future analysis.

The second limitation lies in the assumption that the observed mortality rate ratio *R* ^(obs)^ in 2014 as reported in [ Scheidt-Nave 2019] equals the true rate ratio *R* in 2012. Since the mortality rate ratio is relatively stable [p. 59 in Breslow *et al.*, 1980], the mismatch between the two years is unlikely to impose a problem. However, we cannot assess the difference between the observed and true rate ratio. The main reason is the brief and vague description of the methods to estimate *R* in [ Scheidt-Nave 2019]. For example, it remains unclear how the possible problem of competing risks (contracting diabetes versus dying without diabetes) has been addressed. However, the findings in [ Scheidt-Nave 2019] are consistent with epidemiological surveys in Germany [ Röckl *et al.*, 2017] and with observations from the Danish diabetes register [ Carstensen *et al.*, 2020]. Thus, we think that the assumption *R* ^(obs)^ = *R* is justified.

Apart from these limitations, our findings stress the importance of considering diagnostic accuracy when estimating excess mortality from aggregated data using the method described in Equation (1). In particular the specificity in the younger age-groups should be taken care about.

## Data Availability

### Underlying data

Zenodo: Simulation to study impact of diagnostic accuracy on estimation of excess mortality, http://doi.org/10.5281/zenodo.4300684 [ Brinks *et al.*, 2020].

Zenodo: Estimation of excess mortality from incidence and prevalence: impact of the diagnostic accuracy, http://doi.org/10.5281/zenodo.4302183 [ Brinks *et al.*, 2020].

### Extended data

Zenodo: Extended Data: Impact of diagnostic accuracy on the estimation of excess mortality from incidence and prevalence - simulation study and application to diabetes in German men, http://doi.org/10.5281/zenodo.4434806 [ Brinks *et al.*, 2021].

This project contains the following extended data:

- Detailed derivations of the Equations (1) to (4).

Data are available under the terms of the Creative Commons Attribution 4.0 International license (CC-BY 4.0).
