# Supplementary material for: Impact of diagnostic accuracy on the estimation of excess mortality from incidence and prevalence: simulation study and application to diabetes in German men
Source: F1000Res. 2021 Jan 27;10:49. [Version 1] doi: 10.12688/f1000research.28023.1 (PMC8170531; doi:10.12688/f1000research.28023.1)
Supplement: Peer review history

# Peer review: Reviewer response for version 1

**DOI:** 10.5256/f1000research.30994.r85843

**Published:** 2021-06-07

**Authors:** Bruce Bartholow Duncan

**Affiliations:** 1 Postgraduate Program in Epidemiology and Hospital de Clínicas de Porto Alegre, Universidade Federal do Rio Grande do Sul, Porto Alegre, Brazil

This is an important evaluation of the use of secondary data from a claims database to estimate the sensitivity and specificity of the inclusion in the database of chronic diseases present in the covered population. Secondary data are increasingly being used for disease surveillance, in this case for diabetes mellitus. As they come with error, corrections must frequently be made in analyses. These corrections must be derived and evaluated, as is done here. My comments do not include a verification of the equations presented, which I have no reason to doubt. However, such verification is a task for someone other than myself.

 Major comments:

1. "false positive ratio", I believe, should be "false positive rate".
2. The authors have appropriately alerted that sensitivity and specificity, as used here, are not of a diagnostic test, but rather of the presence of a diagnosis in the claims data. As these terms are applied in a context different from the usual one, I believe that readers would benefit from a bit greater detail, noting that sensitivity is the capacity of the claims system to include in its database all cases of diabetes (whether detected or not) present in those covered by the system and specificity is the capacity to include only true cases of diabetes among those covered. Thus, for example, a covered individual who has diabetes but was never tested and thus never detected would be a false negative, detracting from sensitivity.
3. The horizontal axis of graphs in Figure 3 is the "Relative importance". Please define what this means.
4. At the end of the Results, the authors state: "...which means that roughly 3 in 2000 diagnoses of type 2 diabetes at that age are false positive findings...". As the FPR is being described, the denominator should not be diagnoses of type 2 diabetes, but rather covered individuals truly without diabetes.
5. A major issue for the diabetes epidemiology community is the relative frequency of undiagnosed diabetes, i.e., for every 100 true cases, how many are unknown cases). Some discussion of how to use the approach presented to achieve estimates of the prevalence of undiagnosed diabetes (1-positive predicted value) could increase the relevance of this report (or a future one).
6. Figure 4: Is it possible to trace not only the bounds of the estimated FPR, but also the FPR point estimate at each age?
7. Why are the base-case sensitivity and specificity so high? In terms of sensitivity, the IDF´s 2019 Diabetes Atlas (https://www.diabetesatlas.org/en/) estimates that 24% of those with diabetes in its European region are undiagnosed. A German investigation estimated that between 3 and 9% of adults had undiagnosed diabetes (Tamayo *et al.*, 2014 ^1^). In terms of specificity, the fact that several percent of those who report having diabetes, when tested, are found to have normoglycemia (1-positive predicted value), coupled with the known large within-individual (biologic) variability over time of available means of diagnosis, suggests that specificity is not 99.95%.
8. Is not the greater impact of specificity mainly due to the fact that many more individuals in the population do not have diabetes than do, and thus the specificity is acting on a larger (at younger ages far larger) fraction of the population?
9. The mortality rate ratio of diabetes has declined considerably over recent decades (see: Tables 3 and 4 of Gregg *et al.* (2018 ^2^). However, as you state, the impact of this decline over a 2 year period is likely to be sufficiently small as to not impose a problem.

Minor comments: 

1. Keywords should be reviewed. My understanding is that they should be MeSH terms. Thus, for example,  "lupus" should be "systemic lupus erythematosus".
2. 1st sentence Introduction, better: "...of chronic conditions has become...".
3. Page 4, before "Simulation studies", better:  "...For example, the sensitivity of a code for type 2 diabetes in the claims database in 80 years old...".
4. Last sentence page 4, better: "exemplified" than "motivated".
5. Discussion, second paragraph: I don´t understand what "accurately possible" means.

Additional comments related to specific review questions;

- As I am not fluent in R, I cannot verify that the additional materials include the source data. I imagine not, as the source data must be huge, and initially with personal identifiers.

Is the rationale for developing the new method (or application) clearly explained?

Yes

Is the description of the method technically sound?

Yes

Are the conclusions about the method and its performance adequately supported by the findings presented in the article?

Partly

If any results are presented, are all the source data underlying the results available to ensure full reproducibility?

Partly

Are sufficient details provided to allow replication of the method development and its use by others?

Yes

Reviewer Expertise:

diabetes epidemiology

I confirm that I have read this submission and believe that I have an appropriate level of expertise to confirm that it is of an acceptable scientific standard, however I have significant reservations, as outlined above.

## References

[1] : Regional differences of undiagnosed type 2 diabetes and prediabetes prevalence are not explained by known risk factors. *PLoS One* .2014;9(11) : 10.1371/journal.pone.0113154 e113154 10.1371/journal.pone.0113154 25402347PMC4234669

[2] : Trends in cause-specific mortality among adults with and without diagnosed diabetes in the USA: an epidemiological analysis of linked national survey and vital statistics data. *The Lancet* .2018;391(10138) : 10.1016/S0140-6736(18)30314-3 2430-2440 10.1016/S0140-6736(18)30314-3 29784146

# Peer review: Reviewer response for version 1

**DOI:** 10.5256/f1000research.30994.r85844

**Published:** 2021-06-01

**Authors:** Dianna J. Magliano

**Affiliations:** 1 Department of Diabetes and Population Health, Baker Heart and Diabetes Institute, Melbourne, Vic, Australia; 2 School of Public Health and Preventive Medicine, Monash University, Melbourne, Vic, Australia

This is a modelling analysis which aims to assess the impact of diagnostic accuracy on the estimation of excess mortality from incidence and prevalence using a simulation study. The stimulation study which is developed tests two scenarios: one with a high prevalence setting and the other with a low prevalence setting. The finding is then applied to real diabetes data from claims data from the German Statutory Health insurance. The modelling shows that when estimating excess mortality of diabetes, diagnostic accuracy is very important. Specificity is more important than sensitivity across all age groups, and in particular, specificity in younger people has the greatest impact on the estimated mortality rate ratios.

 Overall, this is a clear and well-presented piece of work. One thing which may be useful is to have some idea of the size of the impact of specificity on the estimation of mortality ratio rate, in comparison to the effect of sensitivity. The authors state that there is a difference between the effect of sensitivity and specificity, but it may be useful for the reader to understand how much of an impact it has.

 My other points are minor and relate to language:

1. The last line of the abstract should be re-written. Starting that sentence with ‘especially’ means the sentence is unclear. You could start with: ‘In particular…’.
2. The first sentence of the introduction could be re written to say: “…chronic diseases are becoming more available.”
3. The heading in the first row of table 1 could be more descriptive. Expand on “setting”. In the actual table heading: insert the word “used” between “settings “and “in”.
4. Table entries of “Lupus” should be written in full.
5. Significant figures in table 1 are not consistent. I do understand why though.
6. Figure 3 should have the panels labelled on figure. “low prevalence” and “high prevalence” or A and B. 

Is the rationale for developing the new method (or application) clearly explained?

Yes

Is the description of the method technically sound?

Yes

Are the conclusions about the method and its performance adequately supported by the findings presented in the article?

Yes

If any results are presented, are all the source data underlying the results available to ensure full reproducibility?

Yes

Are sufficient details provided to allow replication of the method development and its use by others?

Partly

Reviewer Expertise:

Diabetes epidemiology

I confirm that I have read this submission and believe that I have an appropriate level of expertise to confirm that it is of an acceptable scientific standard.

# Peer review: Reviewer response for version 1

**DOI:** 10.5256/f1000research.30994.r85845

**Published:** 2021-05-24

**Authors:** Andreas Wienke

**Affiliations:** 1 Institute of Medical Epidemiology, Biostatistics, and Informatics, Martin-Luther-University Halle-Wittenberg, Halle, Germany

First I would like to congratulate the authors for this excellent paper which examines how limited diagnostic accuracy in terms of sensitivity and specificity affects estimates of excess mortality based on prevalence and incidence data. In the first part relevant formulas from previous work by the authors are given with respect to the relationship between prevalence and incidence on one side and on excess mortality on the other side. Then, based on assumptions about sensitivity and specificity of aggregated data the influence of sensitivity and specificity at different ages in a high and low prevalence situation are investigated by simulations. One key result is that specificity can be obtained without knowledge of the sensitivity in lower age groups. Furthermore, the false positive ratio is investigated and quantified. Finally, the methodology is applied to diabetes 2 data of 35 million men in the German Statutory Health Insurance.  

 The paper is written in a very clear and sound style, I have only very minor remarks:

1. At page 4 the authors state that sensitivity of diagnosing type 2 diabetes in 80 years old people is higher than in 40 years old people. Surprisingly, this is not taken into account in Table 1 where sensitivity is given as 95% for both age groups.
2. In Figure 2 there is no blue line to see because of the coincidence of the simulation and the perfect estimation. It is explained in the text, but should be solved for the figure.
3. Maybe it makes the discussion in the second last paragraph more clear when the authors add (again) that the estimates of R ^(^^obs)^ considered there are based on individual data.

Is the rationale for developing the new method (or application) clearly explained?

Yes

Is the description of the method technically sound?

Yes

Are the conclusions about the method and its performance adequately supported by the findings presented in the article?

Yes

If any results are presented, are all the source data underlying the results available to ensure full reproducibility?

Yes

Are sufficient details provided to allow replication of the method development and its use by others?

Yes

Reviewer Expertise:

biostatistics and epidemiology

I confirm that I have read this submission and believe that I have an appropriate level of expertise to confirm that it is of an acceptable scientific standard.
